# Boron Nitride-Modified Hemp Nanofiber Reinforced Slag-Based Geopolymer Composites: Mechanical, Microstructural and Fire Resistance Performance

**DOI:** 10.3390/polym18111288

**Published:** 2026-05-24

**Authors:** Ahmet Filazi, İsmail Melih Tezcan, Reyhan Akat, Deniz Doğan, Ümit Erdem

**Affiliations:** 1 Department of Construction, Kırıkkale Vocational School, Kırıkkale University, Kırıkkale 71450, Turkey; ahmetfilazi@kku.edu.tr; 2 Department of Civil Engineering Graduate Student, Institute of Science, Kırıkkale University, Kırıkkale 71450, Turkey; ismailmelihtezcan@gmail.com; 3 Department of Architecture, Faculty of Engineering and Architecture, Yozgat Bozok University, Yozgat 66900, Turkey; 4 Faculty of Engineering and Natural Sciences, Kırıkkale University, Kırıkkale 71450, Türkiye; denizdogan@kku.edu.tr; 5 Department of Electronics and Automation, Kırıkkale Vocational School, Kırıkkale University, Kırıkkale 71450, Turkey; umiterdem@kku.edu.tr

**Keywords:** geopolymer, boron nitride, hemp fiber, fire resistance, nanocomposite, electrospinning, sustainable material, GGBFS, SDG 9, SDG 11, SDG 12

## Abstract

This study investigates the mechanical performance, high-temperature resistance, and microstructural characteristics of ground granulated blast furnace slag (GGBFS)-based geopolymer composites reinforced with boron nitride (BN)-modified hemp nanofibers. BN-modified hemp nanofibers (PVA-mBN/Hemp) were produced via electrospinning and incorporated into geopolymer mixtures at varying ratios ranging from 0 to 4 wt%. The effects of nanofiber content on composite properties were evaluated through mechanical testing, ultrasonic pulse velocity (UPV) measurements, and exposure to elevated temperatures (300–1200 °C), supported by SEM-EDS, FTIR, and XRD analyses. The results indicate that low nanofiber additions (0.5–1 wt%) improve flexural strength by up to 15%, although compressive strength is slightly reduced due to increased porosity. UPV measurements confirm the changes in internal structure. At elevated temperatures, nanofiber-reinforced samples exhibit enhanced residual strength compared to the control specimens, particularly at moderate temperatures, whereas significant degradation occurs above 900 °C. Microstructural analyses reveal improved fiber-matrix interaction, reduced crack propagation, and enhanced thermal stability attributed to BN modification. Overall, the incorporation of 0.5–1 wt% BN-modified hemp nanofibers provides an effective balance between mechanical performance and high-temperature resistance, highlighting their potential for use in sustainable and fire-resistant construction materials. **This study contributes to the United Nations Sustainable Development Goals (SDGs), particularly SDG 9 (Industry, Innovation, and Infrastructure), SDG 11 (Sustainable Cities and Communities), and SDG 12 (Responsible Consumption and Production)**.

## 1. Introduction

The construction industry is responsible for approximately 7–8% of global anthropogenic CO_2_ emissions, primarily due to the widespread production of ordinary Portland cement (OPC) [1]. This significant environmental burden has driven the search for low-carbon alternative binders. In this context, geopolymers—inorganic polymers synthesized through the alkaline activation of aluminosilicate precursors such as fly ash, metakaolin, and ground granulated blast furnace slag (GGBFS)—have emerged as a promising sustainable alternative to OPC [1,2]. Compared to OPC, geopolymers can substantially reduce CO_2_ emissions and energy consumption while utilizing industrial by-products [3].

Beyond their environmental benefits, geopolymers exhibit superior engineering properties including high mechanical strength, excellent chemical resistance, and remarkable thermal stability [4,5]. These characteristics make geopolymers attractive for applications requiring fire resistance and structural durability, such as tunnels, high-rise buildings, and industrial facilities. However, despite these advantages, geopolymers inherently display brittle fracture behavior and can lose structural integrity under high-temperature or thermal shock conditions [6]. When exposed to elevated temperatures above 300 °C, geopolymers undergo dehydration of the gel structure, microcrack formation, and subsequent strength loss. Kong and Sanjayan [6] reported that geopolymer paste experiences significant strength degradation above 300 °C due to decomposition of the aluminosilicate gel network. Similarly, previous studies have shown that geopolymer mortars can lose substantial portions of their mechanical strength after exposure to elevated temperatures [7,8].

Fiber reinforcement has been widely adopted as an effective strategy to overcome the brittleness and thermal degradation of geopolymers. Fibers contribute to crack bridging, energy dissipation, and increased fracture toughness [9,10]. Synthetic fibers such as steel, polyvinyl alcohol (PVA), and polypropylene have been extensively studied in geopolymer matrices. Al-Mashhadani et al. [10] reported that steel and PVA fibers increased the flexural strength of fly ash-based geopolymer mortars by 31% and 39%, respectively. Bhutta et al. [9] demonstrated that micro-fibers improved the performance characteristics of geopolymer mortars for repair applications. Dollente et al. [11] showed that PVA and steel fibers significantly enhanced the mechanical strength of geopolymer mortars. However, synthetic fibers are derived from non-renewable petroleum-based resources and have a relatively high environmental footprint.

In recent years, natural fibers have gained increasing attention as sustainable reinforcement materials due to their renewability, low density, good thermal insulation properties, and low cost [12,13]. Among natural fibers, hemp (*Cannabis sativa*) stands out due to its high cellulose content, low density, and excellent specific strength [14,15]. Several studies have investigated the use of hemp fibers in geopolymer composites. Suwan et al. [16] reported that alkali-treated hemp fibers significantly improved the flexural strength and impact resistance of geopolymer mortars, although compressive strength slightly decreased. Narattha et al. [17] observed that hemp fiber addition reduced thermal conductivity while increasing flexural strength and crack resistance. Mostefai et al. [18] demonstrated that hemp fibers could increase the strength of geopolymers through microstructural improvements. Taye et al. [19] showed that hemp fiber reinforcement reduced the brittleness of geopolymers by improving fracture behavior. Simonova et al. [20] investigated crack initiation in geopolymer mortars with hemp fibers and confirmed positive effects on crack control.

However, the application of natural fibers in geopolymer matrices faces two major challenges. First, natural fibers undergo chemical degradation in the highly alkaline environment of geopolymer matrices. Second, the weak fiber-matrix interfacial bonding limits effective load transfer [21,22]. Poletanovic et al. [21] reported that hemp fibers experience significant mass loss and strength reduction after prolonged exposure to alkaline solutions, primarily due to the dissolution of hemicellulose and lignin. Gholampour et al. [22] emphasized that the use of natural fibers in geopolymers requires addressing durability issues related to alkaline degradation. These challenges necessitate the development of fiber surface modification strategies to improve both alkali resistance and interfacial bonding.

Boron nitride (BN), particularly hexagonal boron nitride (h-BN), has emerged as a promising nanomaterial for enhancing the thermal stability and mechanical properties of composite systems. BN exhibits high thermal conductivity, excellent thermal stability up to 1000 °C in air, chemical inertness, and intrinsic flame-retardant properties [23,24]. Liu et al. [23] demonstrated that h-BN nanosheets provided oxidation resistance up to 900 °C in wood coating applications. Dong et al. [24] reported that nano-BN addition improved the aging resistance and reduced the oxidation of fire-resistant coatings at high temperatures. Duan et al. [25] reviewed the properties of h-BN matrix composite ceramics and highlighted their superior high-temperature strength and thermal shock resistance. In geopolymer systems, Alnamel and Baqi [26] showed that BN nanoplatelets improved the physical and mechanical properties of geopolymers. Despite these promising attributes, the use of BN as a fiber surface modifier in natural fiber-reinforced geopolymer composites remains largely unexplored.

Fiber surface modification using BN has been successfully demonstrated in polymer composites. Qiu et al. [27] produced BN nanofibers via electrospinning followed by thermal treatment, achieving controlled fiber morphology. Ahmad et al. [28] showed that h-BN-containing polymer nanofibers exhibited significantly improved thermal stability and mechanical strength. Fang et al. [29] emphasized that the unique combination of high thermal conductivity, mechanical strength, and chemical stability makes BN an ideal component for fiber modification. Akay et al. [30] investigated BN-filled polymer nanofibers produced via electrospinning and confirmed improved properties. However, to the best of the authors’ knowledge, no study has systematically investigated the incorporation of BN-modified hemp nanofibers into GGBFS-based geopolymer matrices for enhanced fire resistance.

A critical review of the literature reveals that while geopolymers, natural fiber reinforcement, and BN modification have been studied independently, no systematic investigation has been conducted on the combined effect of BN-modified hemp nanofibers on the mechanical and fire resistance performance of GGBFS-based geopolymer composites. Specifically, the influence of BN modification on fiber-matrix interfacial bonding, high-temperature phase stability, and post-fire mechanical strength retention remains unknown.

The hypothesis of this study is that the addition of BN-modified hemp nanofibers to GGBFS-based geopolymer mortars will improve high-temperature resistance by: (i) enhancing fiber-matrix interfacial adhesion through BN surface modification, (ii) limiting crack propagation via nanofiber bridging, and (iii) suppressing alkali volatilization and gel decomposition at elevated temperatures due to the thermal stability of BN.

The specific objectives of this study are to produce polyvinyl alcohol (PVA)-based boron nitride-modified hemp (PVA-mBN/Hemp) hybrid nanofibers via electrospinning and optimize the production parameters; to investigate the effect of different nanofiber addition ratios (0%, 0.5%, 1%, 2%, and 4% by weight) on the mechanical properties (compressive and flexural strength) and ultrasonic pulse velocity (UPV) of GGBFS-based geopolymer mortars; to evaluate the high-temperature resistance of the composites after exposure to 300 °C, 600 °C, 900 °C, and 1200 °C; to characterize the microstructural evolution, phase transformations, and chemical changes using SEM-EDS, FTIR, and XRD analyses; and to determine the optimal nanofiber addition ratio that balances ambient-temperature mechanical performance and high-temperature resistance.

Unlike previous studies that have used BN either as a direct additive or only as a surface coating, this study integrates BN into hemp nanofibers via electrospinning combined with TEMPO oxidation and a PVA matrix. Furthermore, while existing works have investigated BN-modified polymer nanofibers or natural fiber-reinforced geopolymers separately, no previous study has systematically incorporated BN-modified hemp nanofibers into GGBFS-based geopolymer composites for combined mechanical and fire resistance performance. Additionally, this study provides a comprehensive evaluation of high-temperature behavior up to 1200 °C with detailed microstructural analyses (SEM-EDS, FTIR, XRD), whereas most previous studies on natural fiber-reinforced geopolymers have focused only on ambient-temperature properties.

This study presents the first systematic investigation of boron nitride (BN)-modified electrospun hemp-based nanofibers as reinforcement in ground granulated blast furnace slag (GGBFS)-based geopolymer composites. Unlike previous studies, the present work integrates nanofiber production via electrospinning, BN surface modification, and comprehensive high-temperature performance evaluation within a unified framework. This combined approach enables a deeper understanding of the coupled effects of nanofiber morphology, surface chemistry, and thermal stability on fiber–matrix interactions. The findings provide new insights into the design of sustainable, fire-resistant geopolymer composites and highlight the potential of bio-based nanofibers for advanced construction applications.

## 2. Materials and Methods

### 2.1. Materials

Sodium hydroxide (NaOH, 98% purity, Merck, Darmstadt, Germany), sodium silicate (Na_2_SiO_3_, 26.48% SiO_2_, 8.28% Na_2_O, 65.24% H_2_O, Merck, Darmstadt, Germany), boron nitride (BN, 99% purity, 50 nm, Nanotech, Turkey), polyvinyl alcohol (PVA, Mw~140.000, Merck, Darmstadt, Germany), 3-aminopropiltrietoksisilan (APTES, Merck, Darmstadt, Germany), 2,2,6,6-tetramethylpiperidine 1-oxyl (TEMPO, 98% purity, Merck, Darmstadt, Germany), sodium bromide (NaBr, analytical grade, Merck, Darmstadt, Germany), sodium hypochlorite (NaClO, commercial, local supplier, Kırıkkale, Turkey), and ethanol (Merck, Darmstadt, Germany) were used as received.

**Figure 1:**
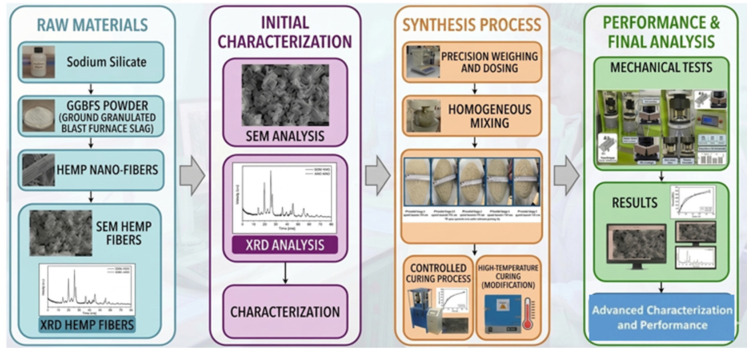
Preparation stage of geopolymer mortar samples.

Ground granulated blast furnace slag (GGBFS) was obtained from Ereğli Iron and Steel Factory (Ereğli, Turkey). CEN standard sand (quartz sand conforming to RILEM-Cembureau standard) was used as fine aggregate. Industrial hemp fibers (Cannabis sativa, 0.5–3.0 mm size, density 1.48–1.50 g/cm^3^) were supplied from a local source in Kırıkkale, Turkey. The properties of all materials are summarized in Table 1 and Figure 1.

### 2.2. Hemp Fiber Modification and Nanofiber Production

h-BN (1.0 g) was dispersed in ethanol (40 mL) by ultrasonication for 30 min, then APTES (0.5 mL) was added and shaken overnight at 60 °C. The modified BN (mBN) was centrifuged and washed with ethanol. Hemp fibers (0.5–3.0 mm) were treated with 5% NaOH for 24 h, neutralized, and dried. TEMPO (0.16 g), NaBr (1.0 g), and NaClO were added at pH 11 for 2 h, then the reaction was terminated with ethanol. The fibers were washed to pH 7 and dried at 40 °C [31] The TEMPO-treated hemp was combined with mBN in ethanol, homogenized at 10,000 rpm for 30 min, and centrifuged to obtain mBN/hemp. A 10 wt% PVA solution was mixed with mBN/hemp (3 wt% BN, 5 wt% hemp) for 24 h, then electrospun at 15 kV, 15 cm distance, and 1.0 mL/h. The nanofibers were heat-treated at 80 °C for 135 min. Six nanofiber types were produced with varying BN (1–5 wt%) and hemp (2–8 wt%) contents.

### 2.3. Preparation of Geopolymer Mortar Specimens

#### 2.3.1. Mix Proportions and Preparation

In this study, sodium hydroxide (NaOH) and sodium silicate (Na_2_SiO_3_) solutions were used as alkaline activator solutions in the preparation of geopolymer mortars. The alkaline activator solution was obtained by mixing NaOH solution prepared at 12 M molarity with Na_2_SiO_3_ solution to ensure effective geopolymerization reaction and achieve high mechanical performance. Considering the literature values commonly used in geopolymer mortar production, the activator/solid ratio was determined as 0.8 and the Na_2_SiO_3_/NaOH ratio as 2.25. It has been reported in various studies that these ratios support geopolymer matrix formation and provide positive effects on mechanical properties.

During mixture preparation, ground granulated blast furnace slag (GGBFS) and standard sand were first mixed homogeneously in dry condition. Then, the prepared alkaline activator solution was added to the mixture, and a homogeneous geopolymer mortar was obtained using a mechanical mixer.

To investigate the effects on the mechanical and high-temperature performance of geopolymer composites, PVA-mBN modified hemp nanofibers were added to the mixture at different ratios. Nanofibers were incorporated into the mortar mixture at 0% (control specimen), 0.5%, 1%, 2%, and 4% of the total mixture weight. Here, “total mixture weight” refers to the combined mass of GGBFS (binder), CEN standard sand (fine aggregate), alkaline activator solution (NaOH + Na_2_SiO_3_), and PVA-mBN/Hemp nanofibers. The nanofiber content is calculated as (mass of nanofibers/total mass of all components) × 100%. The fiber ratios were determined considering the ranges commonly used in fiber-reinforced geopolymer composites in the literature, and it was aimed to comparatively evaluate the effects of different fiber contents on composite properties.

After the prepared specimens were placed into molds, they were cured in an oven at 80 °C and 110 °C for 24 h to accelerate the geopolymerization reaction. The curing temperatures were selected to evaluate the temperature effect on geopolymer matrix formation. The geopolymer mortar mix proportions used in the study are presented in Table 2.

#### 2.3.2. Casting and Curing

The prepared mortar was cast into molds of dimensions 40 × 40 × 160 mm (for mechanical tests) and 50 × 50 × 50 mm (for fire tests). The specimens were cured in a constant temperature-controlled oven at 80 °C and 110 °C for 24 h. After curing, the specimens were demolded and kept at room temperature until the testing ages of 7 and 28 days.

All tests were performed on three replicate specimens per mixture per testing age, and the results are reported as mean ± standard deviation.

### 2.4. Test and Characterization Methods

#### 2.4.1. Fresh Mortar Properties

**Figure 2:**
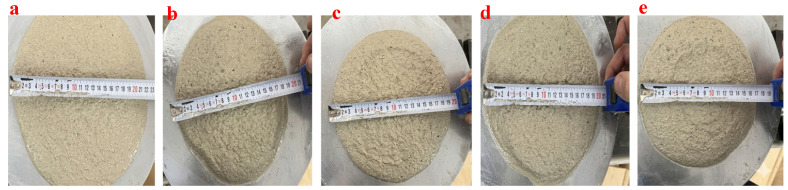
Spread diameter images of (**a**) PVA-mBN/Hemp-G0, (**b**) PVA-mBN/Hemp-G0.5, (**c**) PVA-mBN/Hemp-G1, (**d**) PVA-mBN/Hemp-G2, and (**e**) PVA-mBN/Hemp-G4 specimens.

The workability properties of geopolymer mortars in the fresh state were evaluated by flow table test in accordance with ASTM C1437 standard. The spread diameter of all groups is shown in Figure 2.

#### 2.4.2. Mechanical Tests

**Figure 3:**
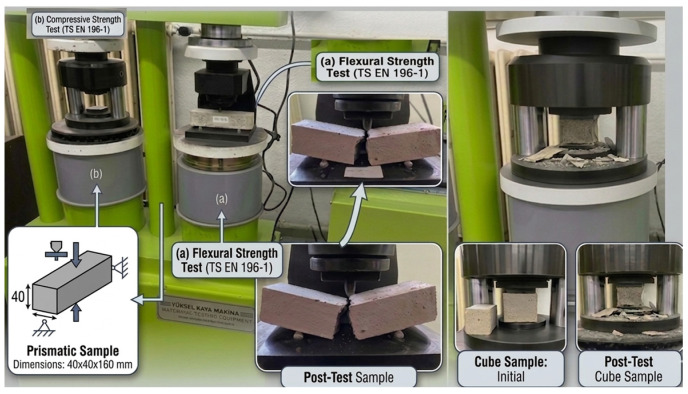
Universal testing machine used in mechanical tests of geopolymer mortars: (**a**) Flexural strength test setup, (**b**) Compressive strength test setup.

The mechanical performance of geopolymer mortar specimens was tested on prismatic specimens of dimensions 40 × 40 × 160 mm in accordance with TS EN 196-1 standard at the end of curing periods of 7 and 28 days. Flexural strength tests were carried out using a three-point bending setup, while compressive strength tests were performed on the half-prisms obtained after the flexural test. The universal testing machine used in the experiments is shown in Figure 3. The universal testing machine has a load cell accuracy of ±0.5% of the indicated value, with a testing speed of 50 N/s for flexural strength and 2.4 kN/s for compressive strength, in accordance with TS EN 196-1.

#### 2.4.3. Ultrasonic Pulse Velocity Test

**Figure 4:**
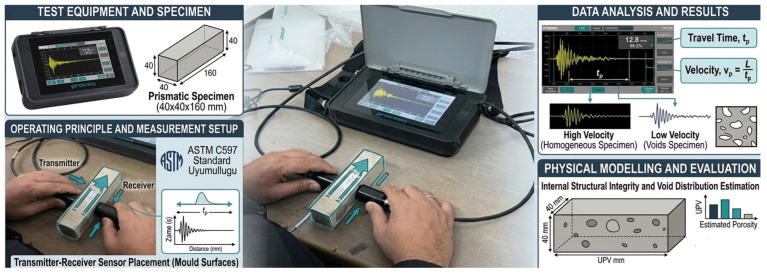
Proceq brand test device used for ultrasonic pulse velocity (UPV) measurement on geopolymer mortar samples.

The internal structural integrity, homogeneity, and void distribution of geopolymer composite specimens were evaluated using the non-destructive ultrasonic pulse velocity (UPV) test. The tests were carried out on prismatic specimens of dimensions 40 × 40 × 160 mm in accordance with ASTM C597 standard. A Proceq brand ultrasonic testing device was used for the measurements. The device has a measurement accuracy of ±0.1 µs and uses transducers with a frequency of 54 kHz, as per the manufacturer’s specifications and ASTM C597 requirements. The device and measurement setup are shown in Figure 4.

#### 2.4.4. High-Temperature Resistance Tests

**Figure 5:**
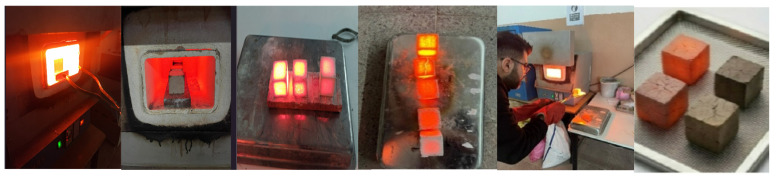
High-temperature performance and fire resistance images of geopolymer composite samples.

To evaluate the high-temperature performance and fire resistance of geopolymer composite specimens, cubic specimens with dimensions of 50 × 50 × 50 mm were subjected to heat treatment in an electric muffle furnace. Starting from room temperature (approximately 23 ± 2 °C), the specimens were heated at a constant rate of 5 °C/min to target temperatures of 300 °C, 600 °C, 900 °C, and 1200 °C. Upon reaching each target temperature, the temperature was maintained for 1 h to ensure uniform thermal distribution throughout the specimens. Subsequently, the specimens were allowed to cool naturally inside the furnace to room temperature to prevent thermal shock. The muffle furnace used in the experiments is shown in Figure 5.

The heating rate of 5 °C/min and holding time of 1 h were selected based on common practices in geopolymer fire resistance literature [6,7,31,32]. This regime allows for gradual thermal exposure, minimizing thermal shock effects while enabling observation of phase transformations and strength degradation. Although standard fire curves (e.g., ISO 834, ASTM E119) involve faster heating rates (up to 100 °C/min) in the early stage, the chosen regime provides a conservative and reproducible laboratory-scale assessment of thermal stability. It is particularly suitable for comparative evaluation of different nanofiber reinforcement ratios rather than exact simulation of real fire scenarios. The electric muffle furnace used in the tests has a temperature accuracy of ±5 °C and a heating rate control accuracy of ±0.5 °C/min, as per the manufacturer’s specifications.

#### 2.4.5. Microstructural and Chemical Characterization

SEM-EDS analyses were performed to examine the microstructure, nanofiber distribution, fiber-matrix interface, and high-temperature-induced changes using a LEO 1430 VP SEM (Carl Zeiss Microscopy, Oberkochen, Germany) equipped with a RÖNTEC QX2 EDS detector (Röntec GmbH, Bruker, Berlin, Germany).

## 3. Experimental Results and Discussion

In this section, the results of experimental studies on PVA nanofiber-reinforced geopolymer mortars containing modified boron nitride (mBN) and hemp fibers subjected to TEMPO oxidation are presented and discussed in light of the literature.

### 3.1. Nanofiber Production and Characterization

#### 3.1.1. Electrospinning Optimization and SEM Analysis

**Figure 6:**
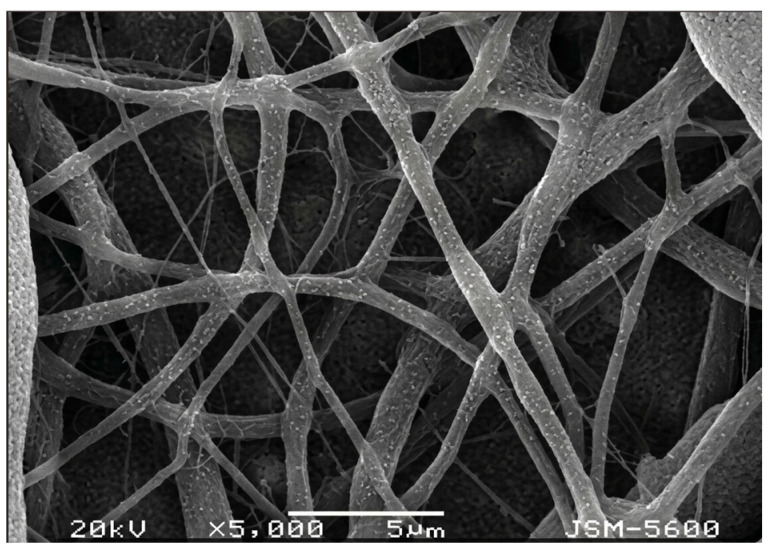
SEM image of PVA-mBN/Hemp nanofibers.

The electrospinning parameters were optimized to achieve bead-free and continuous nanofibers. A voltage of 15 kV was selected as it provided a stable Taylor cone and consistent jet formation. Lower voltages (10–12 kV) resulted in intermittent jet flow and droplet formation, while higher voltages (above 17 kV) caused jet instability and fiber rupture. The working distance was set to 15 cm to allow sufficient solvent evaporation time; shorter distances (10 cm) led to wet fibers and bead formation, whereas longer distances (20 cm) caused fiber thinning and reduced deposition efficiency. The flow rate of 1.0 mL/h was found to be optimal; higher flow rates (1.5–2.0 mL/h) produced thicker fibers with bead defects due to incomplete solvent evaporation, while lower flow rates (0.5 mL/h) resulted in low fiber throughput. Under these optimized conditions, uniform, bead-free PVA-mBN/Hemp nanofibers with diameters ranging from 200 to 800 nm (average 350 ± 50 nm) were successfully produced (Figure 6). The SEM-EDS system (LEO 1430 VP) has an energy resolution of 133 eV at Mn Kα, and the XRD system (Bruker D8 Advance) has a 2θ accuracy of ±0.01°.

#### 3.1.2. FTIR Analysis

**Figure 7:**
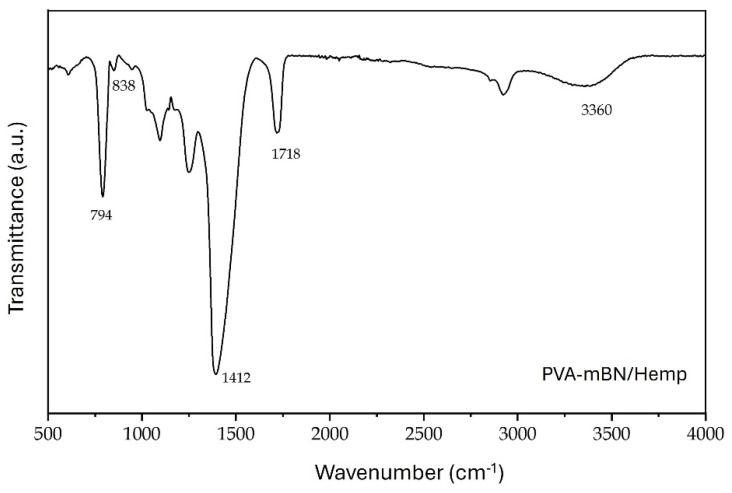
FTIR spectrum of PVA-mBN/Hemp nanofibers.

Fourier-transform infrared (FTIR) spectroscopy was performed to investigate the chemical structure of the PVA-mBN/Hemp nanofibers and the interactions between their constituents. The FTIR spectrum of the PVA-mBN/Hemp nanofibers is presented in Figure 7.

A band at 1718 cm^−1^ (C=O stretching) confirmed successful TEMPO oxidation of hemp fibers, introducing carboxyl groups for stronger BN attachment. The band at 1412 cm^−1^ (C–O ester) confirmed the preserved cellulosic structure.

Characteristic B–N (838 cm^−1^) and B–N–B (794 cm^−1^) bands confirmed successful incorporation of hexagonal BN into the nanofiber structure, consistent with [23,28].

Peak broadening and shifts in O–H and C=O bands indicate hydrogen bonding between PVA and hemp cellulose, improving fiber-matrix interfacial interactions. BN contributes to these interactions, enhancing load transfer and thermal stability, in agreement with [29].

Overall, FTIR confirmed successful formation of a hybrid composite with BN integration, TEMPO oxidation, and improved fiber-matrix interfacial bonding, providing the microstructural basis for enhanced fire resistance and mechanical performance.

#### 3.1.3. Synergistic Effects of TEMPO Oxidation, PVA Electrospinning, and BN Modification

The combined use of TEMPO oxidation, PVA electrospinning, and BN modification creates a synergistic effect that significantly enhances fiber-matrix interfacial bonding and thermal stability, rather than acting as independent contributions.

TEMPO oxidation introduces carboxyl groups (–COOH) onto the hemp fiber surface, increasing surface polarity and chemical reactivity. This functionalization enables stronger hydrogen bonding and covalent interactions with the PVA matrix and the geopolymer matrix. The improved surface chemistry also provides more anchoring sites for BN particles.

PVA electrospinning serves as a carrier matrix that ensures homogeneous distribution of BN particles and hemp nanofibers within the composite. The PVA matrix forms a continuous bridging network throughout the geopolymer, facilitating stress transfer across the fiber-matrix interface. Additionally, PVA acts as a protective layer for hemp fibers against alkaline degradation, which is a critical issue for natural fibers in geopolymer environments.

BN modification provides thermal stability (up to 1000 °C in air), chemical inertness, and intrinsic flame-retardant properties. When integrated into the nanofiber structure, BN creates a thermally stable barrier phase at the fiber-matrix interface. This barrier delays heat transfer, suppresses alkali volatilization, and prevents premature degradation of both the fibers and the geopolymer gel at elevated temperatures.

The synergy among these three components results in:

(i) enhanced fiber-matrix adhesion through combined chemical (TEMPO) and physical (PVA) mechanisms,
(ii) improved thermal stability through BN’s barrier effect
(iii) reduced crack propagation through nano-scale fiber bridging.

Consequently, the nanofiber-reinforced geopolymer composites exhibit superior high-temperature resistance compared to what would be achieved by any of these modifications individually.

### 3.2. Geopolymer Mortar Optimization

#### 3.2.1. Effect of NaOH Molarity on Workability

**Figure 8:**
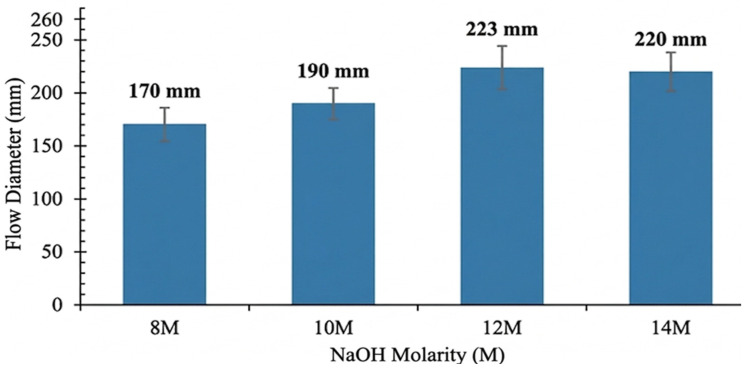
Flow diameter values at different NaOH molarities.

The workability of geopolymer mortars was evaluated at NaOH molarities of 8 M, 10 M, 12 M, and 14 M using a flow table test (Figure 8).

The results presented in Figure 8 indicate that NaOH molarity has a significant influence on the workability of geopolymer mortars. The flow diameter increased from 170 mm at 8 M to 190 mm at 10 M (an increase of 11.8%), and further to 223 mm at 12 M, corresponding to a total increase of 31.2% compared to 8 M. This trend suggests that higher NaOH concentrations enhance dissolution rates, leading to a more fluid matrix. However, increasing the molarity to 14 M resulted in a slight decrease in flow diameter to 220 mm (−1.3%), which can be attributed to excessive OH^−^ concentration accelerating geopolymerization and causing early gel formation. Therefore, optimum workability was achieved at 12 M NaOH. These findings are consistent with the literature, which generally reports an optimal NaOH molarity range of 8–14 M. Previous studies have shown that increasing molarity up to 12 M improves workability, microstructure, and mechanical performance, with 12 M often identified as the optimum level.

In conclusion, NaOH molarity is a critical parameter governing fresh-state workability. At 12 M, sufficient alkalinity ensures effective dissolution while maintaining balanced reaction kinetics, facilitating homogeneous fiber dispersion and improving the overall microstructural integrity and mechanical performance of the composite.

#### 3.2.2. Effect of Optimum Curing Temperature and NaOH Molarity on the Flexural Strength of Specimens

**Figure 9:**
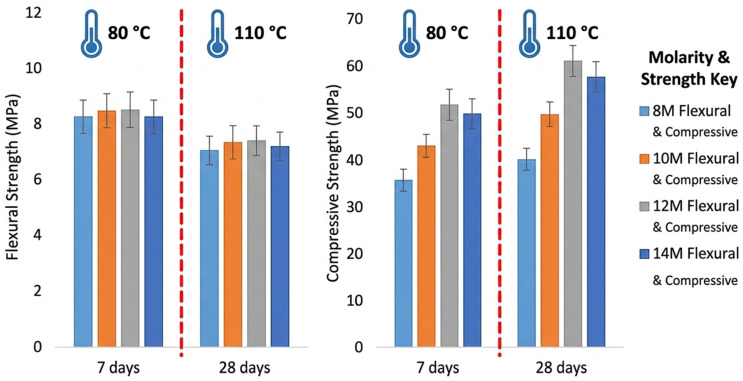
7-day and 28-day flexural strength results of geopolymer mortar samples cured at 80 and 110 °C.

Preliminary tests were conducted on fiber-free geopolymer mortars to determine the optimal NaOH molarity and curing temperature. Mortars prepared with 8 M, 10 M, 12 M, and 14 M NaOH were cured at 80 °C and 110 °C, and tested at 7 and 28 days. The 7-day and 28-day flexural strength results of geopolymer mortar samples obtained in this study are shown in Figure 9.

Preliminary tests were conducted on fiber-free geopolymer mortars to determine the optimum NaOH molarity and curing temperature. Mortars prepared with 8 M, 10 M, 12 M, and 14 M NaOH solutions were cured at 80 °C and 110 °C and tested after 7 and 28 days. Specimens cured at 80 °C exhibited higher and more stable mechanical performance compared to those cured at 110 °C. For 12 M NaOH, flexural strength values were 8.5 MPa (7 days) and 9.7 MPa (28 days) at 80 °C, whereas the corresponding values at 110 °C were 6.5 MPa and 7.6 MPa, respectively. This behavior is attributed to the more controlled geopolymerization process at 80 °C, while excessive water evaporation and microcrack formation at 110 °C adversely affected strength development [32]. In addition, compressive strength increased with NaOH molarity up to 12 M, followed by a slight reduction at 14 M due to accelerated reaction kinetics and heterogeneous microstructure formation. Therefore, a NaOH molarity of 12 M and a curing temperature of 80 °C were selected as the optimum conditions for nanofiber-reinforced geopolymer production.

#### 3.2.3. Ultrasonic Pulse Velocity (UPV) and Compressive Strength Values of Geopolymer Composites Containing Different Ratios of PVA-mBN/Hemp Nanofiber Reinforcement

The internal structural integrity of the composites was assessed through ultrasonic pulse velocity (UPV) measurements (Table 3). With increasing nanofiber content from 0 to 4 wt%, the UPV values (40 mm) decreased markedly from 2.39 to 1.53 km/s, accompanied by a substantial decline in compressive strength from 40.22 to 8.60 MPa. This behavior is attributed to the combined effects of increased pore formation, inadequate fiber dispersion, and nanofiber agglomeration, which disrupt matrix continuity and hinder effective stress transfer. While the incorporation of 0.5–1 wt% nanofibers led to an improvement in flexural strength due to enhanced crack-bridging mechanisms, both compressive strength and UPV values remained significantly lower than those of the control mixture. These findings indicate a fundamental trade-off between enhanced ductility and reduced matrix densification, limiting the load-bearing capacity of the composites.

**Figure 10:**
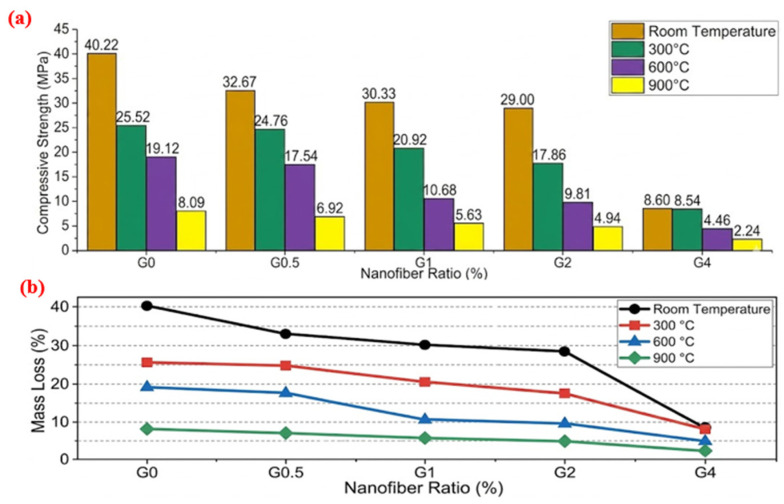
Effect of nanofiber ratio and high temperature (300 °C, 600 °C, and 900 °C) on the geopolymer composites: (**a**) compressive strength and (**b**) mass loss.

Although qualitative SEM analysis (Figure 10) suggests nanofiber agglomeration at higher contents (≥2 wt%), quantitative assessment of fiber dispersion (e.g., dispersion index or image analysis) was beyond the scope of this study. Nevertheless, the systematic and significant decreases in UPV values (from 2.39 to 1.53 km/s at 40 mm) and compressive strength (from 40.22 to 8.60 MPa) with increasing nanofiber content (Table 3) provide indirect quantitative evidence of increased porosity and non-uniform fiber distribution. These results are consistent with the agglomeration effects reported in the literature for fiber-reinforced geopolymers [9,10,21]. Quantitative porosity measurements (e.g., mercury intrusion porosimetry) and detailed characterization of the interfacial transition zone (ITZ) were beyond the scope of this study. However, the systematic decrease in UPV values (Table 3) and SEM observations of fiber-matrix debonding at higher nanofiber contents (≥2 wt%) indirectly support the interpretation that excessive fiber loading increases porosity and creates weak ITZ regions, leading to compressive strength loss. Future work should include these quantitative analyses to further elucidate the mechanisms.

#### 3.2.4. High-Temperature Resistance Performance

To evaluate the behavior of geopolymer composite samples under high temperatures, specimens that had completed the 28-day curing period were exposed to 300 °C, 600 °C, 900 °C, and 1200 °C. At each temperature level, the samples were held for 1 h and then subjected to compressive strength tests after cooling to room temperature. At 1200 °C, all specimens exhibited complete structural collapse, making post-fire mechanical testing impossible. This behavior is attributed to extensive melting and vitrification of the geopolymer matrix. According to the literature, at temperatures above 1000 °C, the amorphous N–A–S–H and C–A–S–H gels transform into crystalline phases such as nepheline, anorthite, and wollastonite, accompanied by the formation of a liquid (glassy) phase that causes severe shrinkage, macro-cracking, and loss of cohesion [6,25,32]. The presence of a glassy layer on the surface of the 1200-exposed samples (Figure 6) supports this vitrification mechanism. Even with BN-modified hemp nanofibers, the thermal stability of the composite is insufficient to maintain structural integrity at this extreme temperature, confirming 1200 °C as the practical upper service limit for GGBFS-based geopolymer systems.

The effect of nanofiber ratio and high temperature values was investigated in this study. The results obtained are shown in Figure 10. The highest compressive strength for all mixtures was observed at 300 °C, attributed to continued geopolymerization and matrix densification. At 600 °C and 900 °C, strength gradually decreased due to water evaporation, microcracking, and gel degradation. Increasing nanofiber content (G0 to G4) led to lower strength at all temperatures due to non-homogeneous distribution, agglomeration, and weak fiber-matrix adhesion. Weight and mass loss increased with temperature and nanofiber content, indicating poor thermal stability of nanofibers at high temperatures.

## 4. Microstructure Analyses

### 4.1. SEM-EDS Analyses

#### 4.1.1. Microstructural and Elemental Analysis of Control Samples

##### Control Sample Subjected to 300 °C Heat Treatment

**Figure 11:**
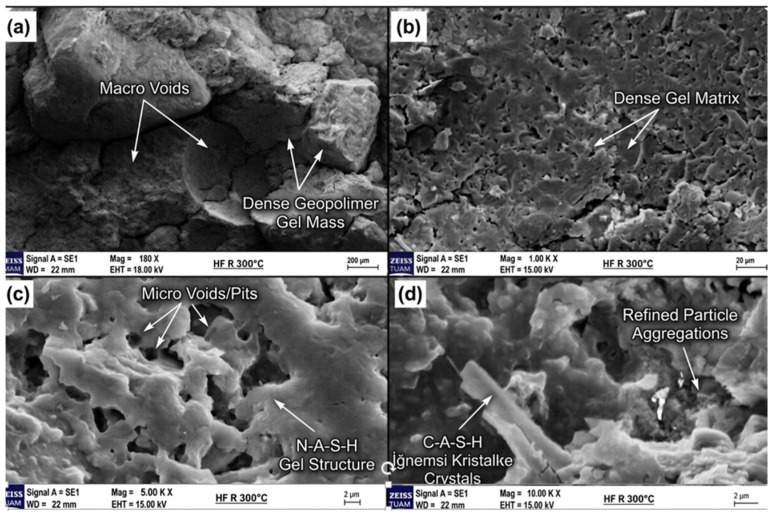
SEM images of the control sample at 300 °C: (**a**) macro voids and dense gel mass; (**b**) dense gel matrix; (**c**) N-A-S-H gel and needle-like C-A-S-H crystals (5000×); (**d**) refined particle aggregations (5000×).

Figure 11 SEM image of the control sample after 300 °C heat treatment shows a heterogeneous microstructure with macro/micro voids, a dense gel matrix, and coexistence of needle-like C-A-S-H crystals and N-A-S-H gel.

**Figure 12:**
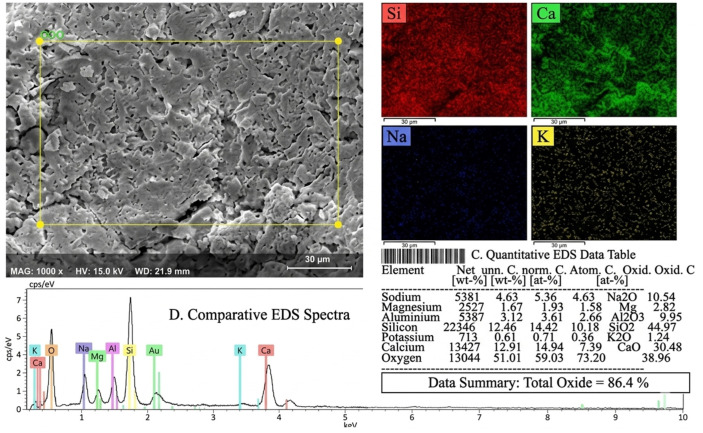
EDS image of the control sample subjected to 300 °C heat treatment.

Figure 12 EDS analysis of the control sample at 300 °C reveals an elemental composition of 59.03% O, 14.94% Ca, 14.42% Si, 5.36% Na, 3.61% Al, and oxide-based contents of 44.97% SiO_2_, 30.48% CaO, and 10.54% Na_2_O, confirming a hybrid binder system with coexisting C-A-S-H and N-A-S-H gels.

##### Control Sample Subjected to 600 °C Heat Treatment

**Figure 13:**
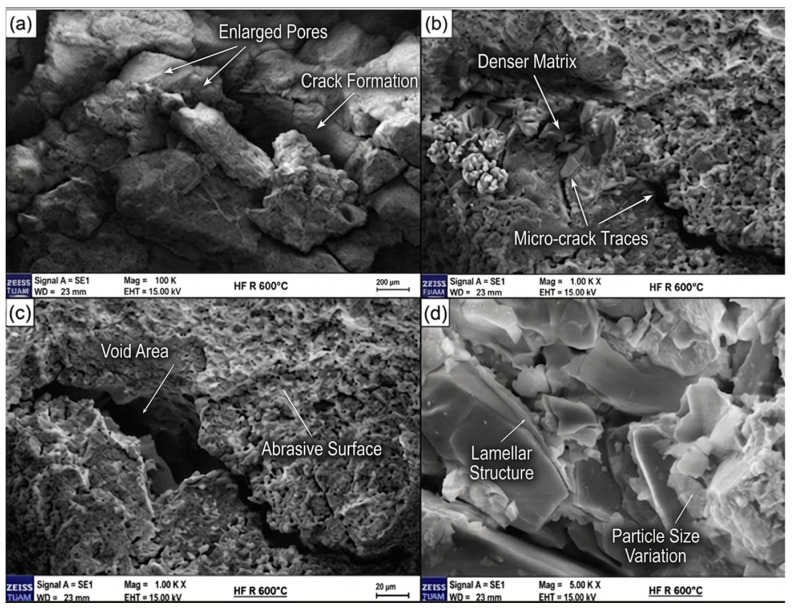
SEM images of the control sample at 600 °C: (**a**) enlarged pores and cracks; (**b**) denser matrix with micro-crack traces; (**c**) void area and abrasive surface; (**d**) lamellar structure with particle size variation.

**Figure 14:**
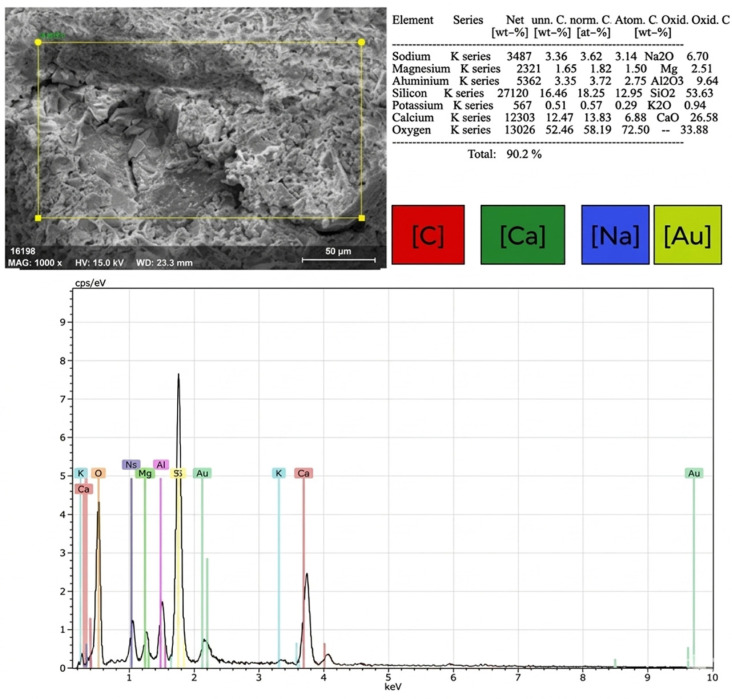
EDS analysis results of the control sample subjected to 600 °C heat treatment.

Figure 13 SEM images of the control sample at 600 °C show expanded macro voids, microcracks, and a layered morphology, indicating that the needle-like C-A-S-H and N-A-S-H gels observed at 300 °C have transformed due to dehydration and phase changes, leading to shrinkage-induced cracking. Also, EDS analysis results of the control sample subjected to 600 °C heat treatment were shown in Figure 14.

##### Control Sample Subjected to 900 °C Heat Treatment

**Figure 15:**
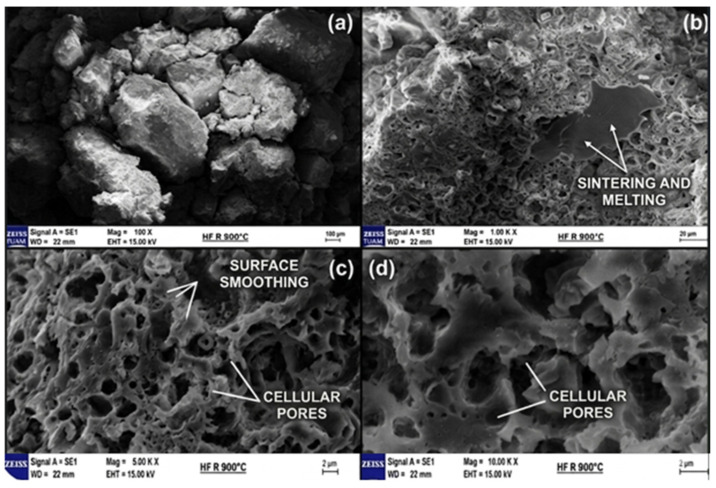
SEM images of the control sample at 900 °C: (**a**) sintering and melting regions (100×); (**b**) surface smoothing (1.00 KX); (**c**) cellular pores (5.00 KX); (**d**) detailed cellular pores (10.00 KX).

Figure 15 SEM images of the control sample at 900 °C reveal sintering, a cellular pore structure, and transformation of amorphous C-A-S-H/N-A-S-H gels into crystalline phases, indicating a ceramic-like character.

**Figure 16:**
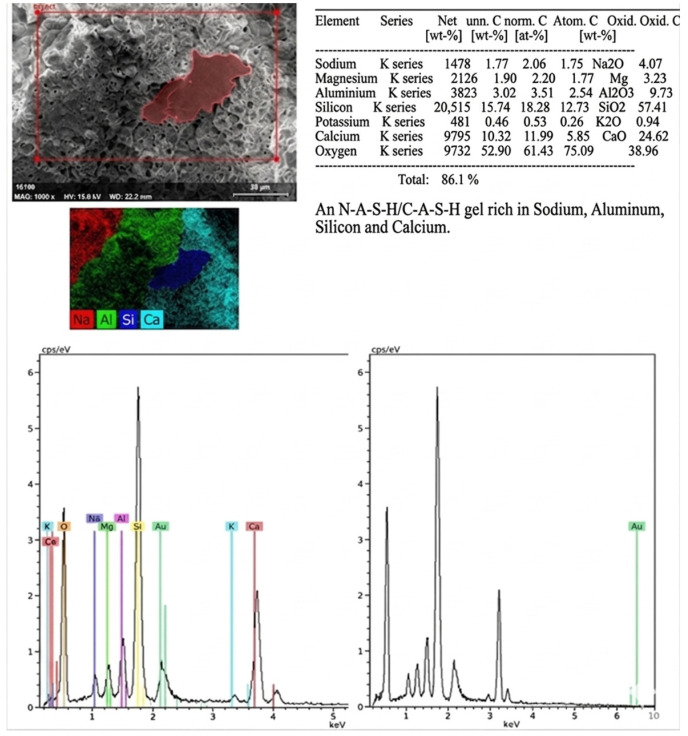
EDS image of the control sample subjected to 900 °C heat treatment.

Figure 16 EDS analysis of the control sample at 900 °C shows 61.43% O, 18.28% Si, 11.99% Ca, and significant sodium loss (2.06% Na), confirming the transformation of amorphous N-A-S-H/C-A-S-H gels into crystalline phases and the evolution toward a ceramic-like structure.

#### 4.1.2. Microstructural and Elemental Analysis of Boron Nitride-Modified Hemp Nanofiber Reinforced Samples

##### BN-Modified Hemp Nanofiber Reinforced Sample Subjected to 300 °C Heat Treatment

**Figure 17:**
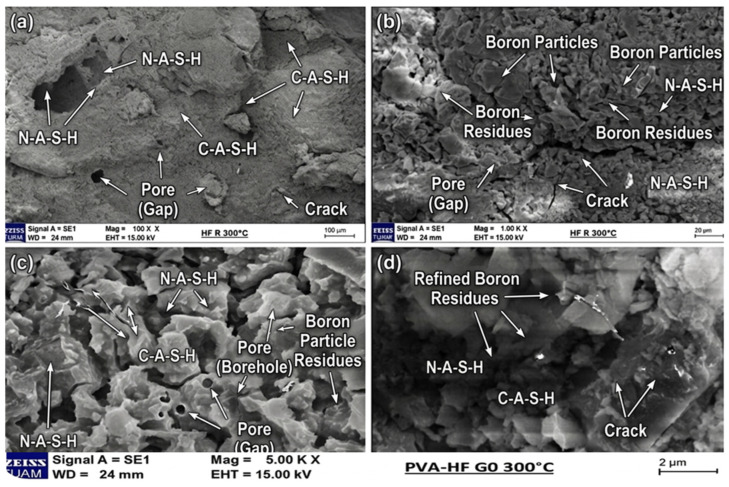
SEM images of BN-modified sample at 300 °C: (**a**) N-A-S-H/C-A-S-H gels with B particles (100×); (**b**) B residues with pore/crack (1.00 KX); (**c**) refined B residues (5.00 KX); (**d**) detailed B residues (5.00 KX, 2 µm).

The SEM images in Figure 17 reveal the effects of nanofiber reinforcement and boron nitride modification on the microstructure.

Compared to the control sample (Figure 11), hemp nanofibers are homogeneously distributed within the matrix, and good fiber-matrix interfacial adhesion is observed. Thanks to BN modification, the thermal stability of nanofibers increased, allowing them to maintain their structural integrity at 300 °C.

At low magnifications, macro voids are reduced in size, and the matrix appears more homogeneous. At high magnifications, hemp nanofibers create a crack-bridging effect, preventing crack propagation and forming fiber-reinforced zones around micro voids. Due to BN modification, a thin BN layer is visible on nanofiber surfaces, which improves fiber-matrix interfacial adhesion.

The N-A-S-H gel structure and needle-like C-A-S-H crystals observed in the control sample also form the main matrix here, but exhibit a denser and more regular morphology due to the presence of hemp nanofibers. Nanofibers contribute to material toughness by stopping or redirecting crack propagation, creating energy dissipation mechanisms.

**Figure 18:**
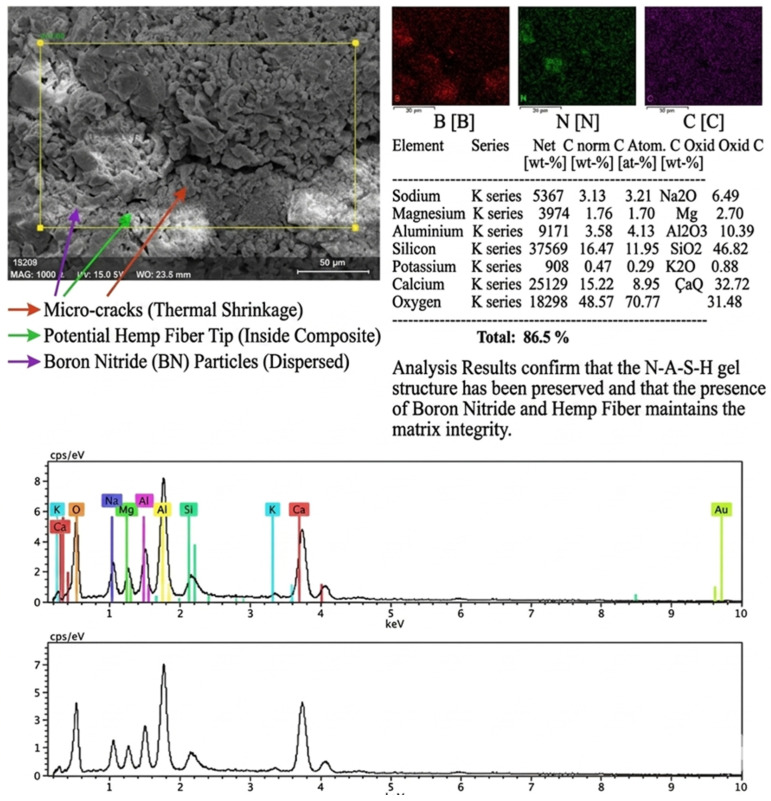
EDS image of the BN-modified hemp nanofiber reinforced sample subjected to 300 °C heat treatment.

Figure 18 EDS analysis of the BN-modified sample at 300 °C confirms the presence of B, N, and C, indicating successful BN coating on nanofibers, while the main oxides (SiO_2_, CaO, Al_2_O_3_, Na_2_O) remain similar to the control, with a more homogeneous and less porous microstructure.

##### BN-Modified Hemp Nanofiber Reinforced Sample Subjected to 600 °C Heat Treatment

**Figure 19:**
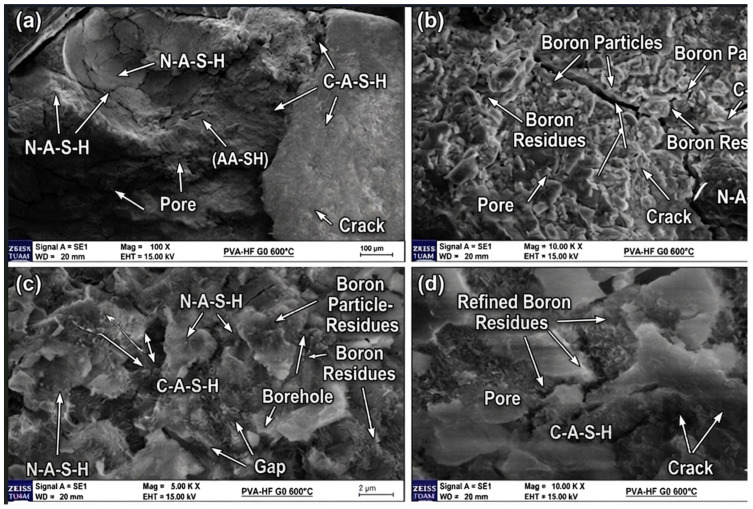
SEM images of BN-modified sample at 600 °C: (**a**) N-A-S-H/C-A-S-H gels with B particles and cracks (100×); (**b**) B residues with pore/crack (10.00 KX); (**c**) refined B residues (5.00 KX, 2 µm); (**d**) B residues and crack formations (10.00 KX).

The SEM images in Figure 19 reveal the effects of high temperature on the microstructure and the behavior of reinforcement elements under these conditions.

At low magnification (a), N-A-S-H and C-A-S-H gels are clearly observed within the matrix. Similar to the control sample (600 °C), crack formations and void structures are present. However, thanks to nanofiber reinforcement and BN modification, crack density and sizes are partially reduced.

At medium magnifications (b), boron particles and boron residues are observed dispersed within the matrix. These particles are boron-containing phases originating from BN modification that remain in the structure at high temperature. These boron residues exist as a secondary phase and likely contribute to thermal stability. Additionally, crack propagation slows or stops around boron particles, indicating that boron-containing phases play a role in crack deflection or arrest mechanisms.

At high magnifications (c and d), void structures between N-A-S-H and C-A-S-H gels and boron particles concentrated around these voids are observed. Image (d) shows the relationship between refined boron residues and boron particle thickness. Boron-containing phases are dispersed at the nanometer scale and form layers in some regions.

Expanded pores and crack formations, prominent in the control sample, are relatively less intense in the BN-modified hemp nanofiber reinforced sample. Nanofibers and boron particles exhibit a crack-arresting effect. “Deep microcracks (600 °C thermal shrinkage)” confirms the presence of shrinkage-induced cracks. “Possible hemp fiber void (within composite)” suggests that hemp fibers may have partially degraded at high temperature, leaving voids.

**Figure 20:**
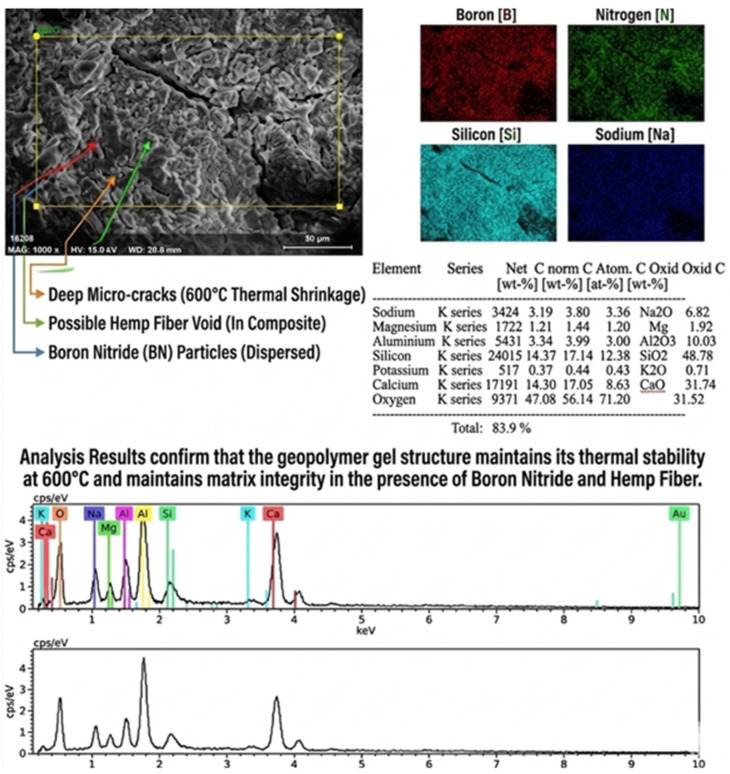
EDS image of the BN-modified hemp nanofiber reinforced sample subjected to 600 °C heat treatment.

Figure 20 EDS analysis confirms BN modification (B and N presence) in the sample at 600 °C. Boron particles dispersed in the matrix prevent crack propagation and enhance thermal stability, while the increased CaO ratio indicates promoted C-A-S-H formation, resulting in better structural integrity than the control.

##### BN-Modified Hemp Nanofiber Reinforced Sample Subjected to 900 °C Heat Treatment

**Figure 21:**
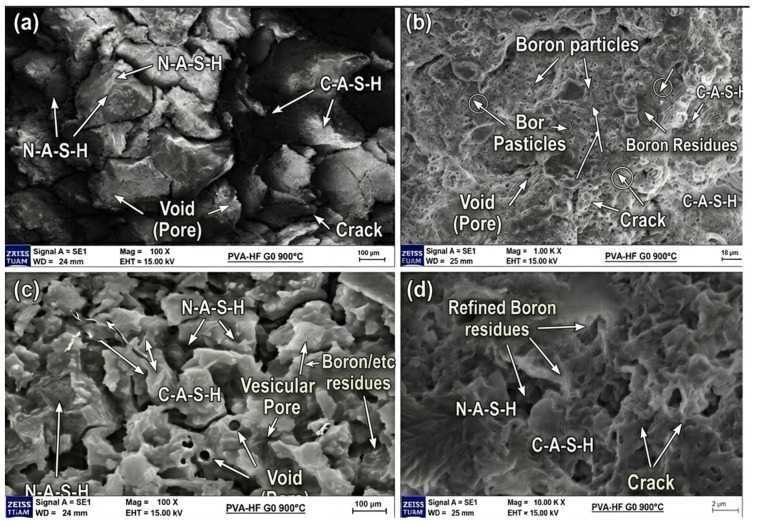
SEM images of BN-modified sample at 900 °C: (**a**) N-A-S-H/C-A-S-H gels with voids and cracks (100×); (**b**) B particles and residues with cracks (1.00 KX); (**c**) vesicular residues with pores/voids (100×); (**d**) refined B residues (10.00 KX).

The SEM images in Figure 21 reveal the profound microstructural transformations caused by high temperature and the behavior of reinforcement elements under these conditions.

At low magnification (a), N-A-S-H and C-A-S-H gels are still present within the matrix, and sintering effects observed in the control sample (900 °C, Figure 18) are also evident. However, due to BN modification and hemp nanofiber reinforcement, the microstructure exhibits a more complex morphology. Void structures and crack formations show different distribution and morphology compared to the control sample.

At medium magnifications (b), boron particles and boron residues are clearly visible dispersed within the matrix. These particles maintain their structural integrity at 900 °C, similar to the 600 °C sample, and persist as a secondary phase. These boron residues remain stable without being affected by the sintering process at high temperature. Additionally, crack propagation changes direction or stops around boron particles, indicating that boron-containing phases play a role in crack arrest mechanisms even at high temperatures.

At high magnifications (c and d), void structures between N-A-S-H and C-A-S-H gels and boron residues concentrated around these voids are observed. Image (d) shows refined boron residues and their distribution between N-A-S-H and C-A-S-H gels. Boron-containing phases are dispersed at the nanometer scale and remain stable within the sintered structure.

While sintering and surface smoothing are observed in both samples, the BN-modified sample exhibits more heterogeneous sintering due to the presence of boron particles. The regular cellular pore structure observed in the control sample becomes more complex in this sample due to boron particles and voids from hemp fiber pyrolysis. The matrix structure, which homogenized with sintering in the control sample, exhibits a more heterogeneous phase distribution in this sample due to the presence of boron particles.

**Figure 22:**
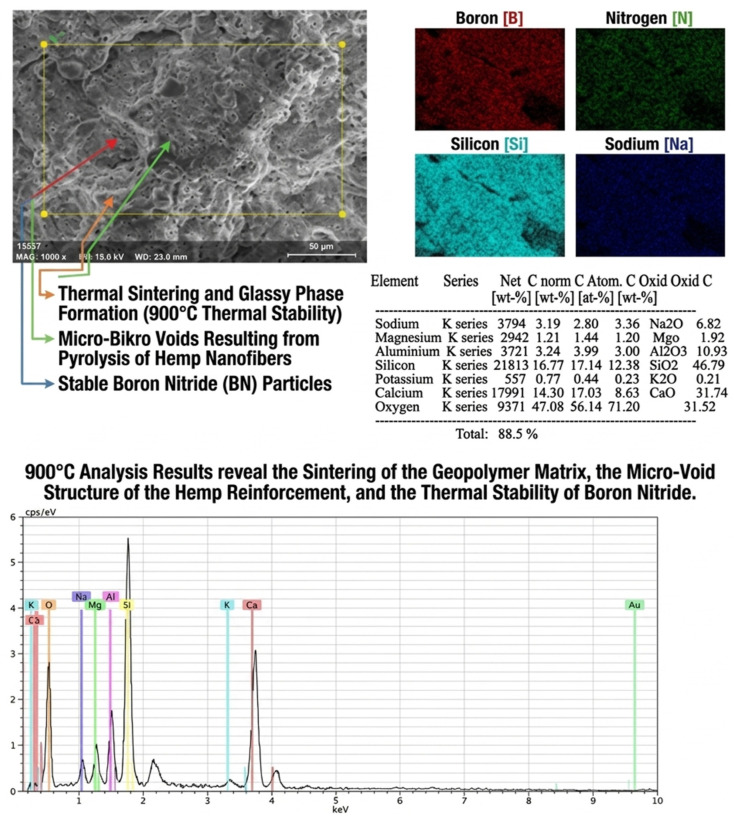
EDS image of the BN-modified hemp nanofiber reinforced sample subjected to 900 °C heat treatment.

Figure 22 EDS analysis of the BN-modified sample at 900 °C confirms that BN particles remain stable, prevent crack propagation, and reduce alkali/calcium loss compared to the control, while microvoids from hemp pyrolysis do not disrupt matrix integrity due to BN modification.

### 4.2. XRD Analyses

#### 4.2.1. XRD Analysis of Samples Subjected to 300 °C Heat Treatment

XRD diffractograms of all specimens showed a broad amorphous halo in the 2θ range of 20–35°, indicating that the geopolymer matrix largely preserved its amorphous gel structure at 300 °C. Addition of 0.5 wt% nanofibers slightly increased the amorphous halo intensity. At 1 wt%, minor peak shifts (2θ ≈ 26° to 25.8°) were observed, attributed to matrix–fiber interfacial microstresses. The highest amorphous halo density was achieved at 2 wt% nanofibers. At 4 wt%, slight narrowing of the amorphous halo and sharpening of crystalline peaks suggested fiber agglomeration and localized crystallization.

**Figure 23:**
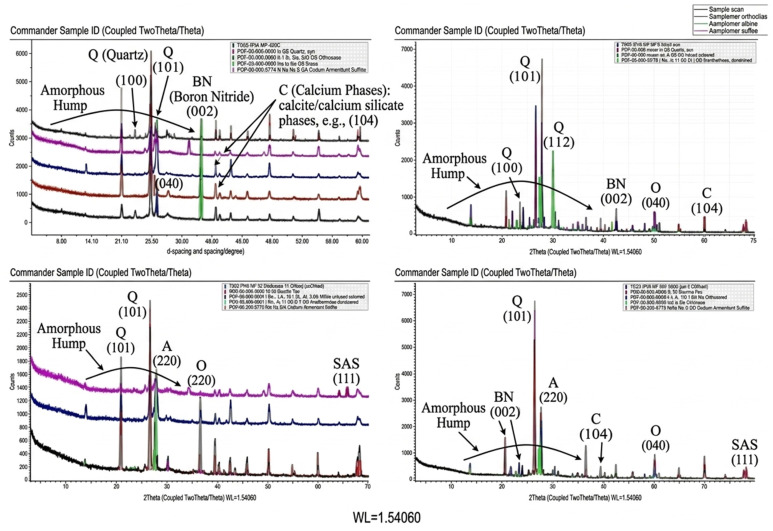
XRD patterns of geopolymers with different compositions subjected to heat treatment at 300 °C.

The XRD patterns presented in Figure 23 illustrate the structural characteristics of geopolymer samples at a relatively low temperature. A broad amorphous hump observed in the 2θ range of 20–35° confirms that the geopolymer matrix largely retains its fundamental amorphous structure at 300 °C. Quartz (Q) was identified as the dominant crystalline phase, with reflections corresponding to the (101), (100), and (112) planes. Additionally, reflections attributable to calcite or calcium silicate phases (e.g., (104) plane) and sulfur-containing phases (SAS (111)) were also detected. In the reinforced samples, a peak corresponding to the (002) plane of the hexagonal boron nitride (BN) structure was observed, indicating that BN maintains its structural stability at 300 °C. Compared to the data obtained at 600 °C, the 300 °C patterns exhibit lower crystallization intensity and represent the early thermal stage of geopolymerization.

#### 4.2.2. XRD Analysis of Samples Subjected to 600 °C Heat Treatment

XRD diffractograms showed a decrease in the amorphous halo intensity (2θ ≈ 26–32°) and an increase in quartz and silicate peaks for the control sample, indicating partial crystallization. Addition of 0.5–1 wt% nanofibers improved amorphous structure preservation with slight peak shifts (2θ ≈ 25.8–26°), attributed to BN modification and enhanced interfacial interactions. At 2 wt%, the amorphous halo remained distinct with limited crystalline peaks, suggesting optimal fiber content. At 4 wt%, sharper crystalline peaks and a weakened amorphous halo indicated fiber agglomeration and localized degradation.

**Figure 24:**
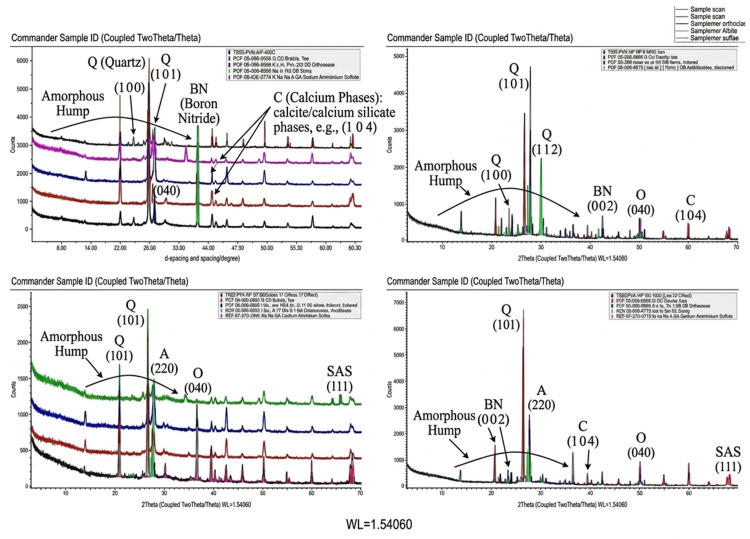
XRD patterns of geopolymer samples subjected to heat treatment at 600 °C.

Figure 24 XRD patterns of geopolymer samples after heat treatment at 600 °C. A broad amorphous hump (20–35° 2θ) indicates partial preservation of the gel structure, while quartz (Q) and BN (002) peaks are observed.

#### 4.2.3. XRD Analysis of Samples Subjected to 900 °C Heat Treatment

**Figure 25:**
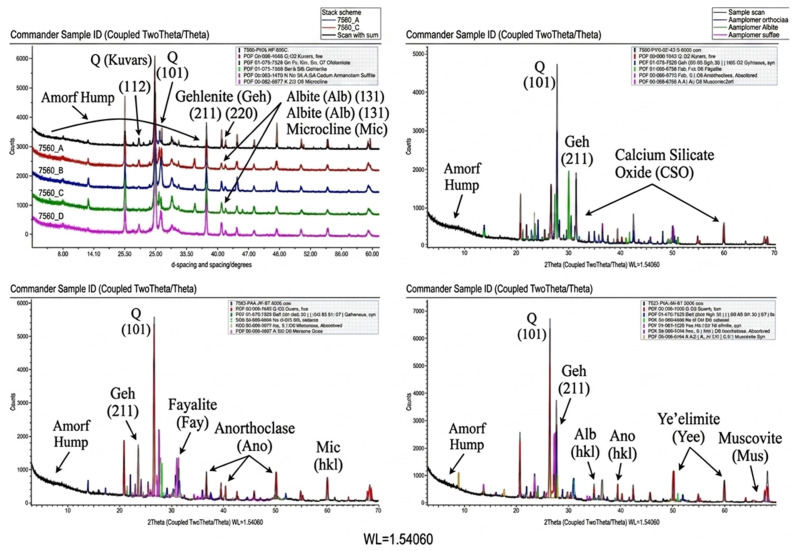
XRD patterns of geopolymers with different compositions subjected to heat treatment at 900 °C.

XRD diffractograms showed (Figure 25) a significant decrease in the amorphous halo intensity (2θ ≈ 26–32°) and a marked increase in crystalline peaks (quartz, feldspar, silicates), indicating that the amorphous gel structure was largely lost at 900 °C. Partial preservation of the amorphous structure was observed only at 1–2 wt% nanofiber addition, while 4 wt% addition increased crystallization and agglomeration. BN modification provided limited thermal stabilization, but complete preservation of the polymeric structure was not achieved at this temperature.

## 5. Conclusions

In this study, the effects of boron nitride (BN)-modified electrospun hemp nanofibers (PVA-mBN/Hemp) at contents of 0–4 wt% on the mechanical properties, high-temperature resistance, and microstructural evolution of GGBFS-based geopolymer mortars were systematically investigated. The main conclusions are summarized as follows:

- The optimum production parameters were determined as 12 M NaOH molarity and an 80 °C curing temperature. In the preliminary optimization stage, the fiber-free reference mixtures prepared under these conditions achieved 28-day compressive and flexural strengths of 63.3 MPa and 9.72 MPa, respectively. However, the control mixture (G0) used in the nanofiber-containing series exhibited a reduced compressive strength of 40.22 ± 1.12 MPa owing to modifications in the mix design required for nanofiber incorporation.
- Electrospun PVA-mBN/Hemp nanofibers exhibited uniform, bead-free morphology with diameters ranging from 200 to 800 nm. FTIR results confirmed successful BN incorporation through the presence of characteristic B–N and B–N–B bonds.
- The incorporation of 0.5–1 wt% nanofibers enhanced flexural strength by up to 15%, primarily due to improved crack-bridging capability and enhanced fiber–matrix interfacial interactions. However, compressive strength did not exhibit comparable improvement, indicating a divergence in load-bearing and crack-resistance mechanisms.
- Higher nanofiber contents (≥2 wt%) led to reduced mechanical performance due to poor dispersion, increased viscosity, and fiber agglomeration, which adversely affected matrix densification.
- At elevated temperatures, the 0.5 wt% nanofiber-reinforced samples demonstrated improved strength retention compared to the control, particularly at 300 °C and 600 °C, indicating enhanced thermal resistance. The temperature range of 300–600 °C was identified as the critical region associated with the onset of C–A–S–H gel degradation.
- At 900 °C, all samples experienced severe strength loss (>80%), while at 1200 °C, complete structural collapse was observed, indicating the upper thermal limit of the geopolymer system regardless of fiber reinforcement.
- Microstructural analyses (SEM, EDS, and XRD) revealed that BN-modified nanofibers contributed to improved matrix integrity at intermediate temperatures by reducing elemental loss and delaying microstructural degradation. However, at higher temperatures, sintering and pore coarsening dominated the structural response.
- The overall behavior of the composites is governed by the synergistic effects of BN modification, TEMPO-induced surface functionalization, and PVA-assisted fiber bridging. BN acts as a thermally stable barrier phase, promoting heat dissipation and crack deflection, while TEMPO oxidation enhances interfacial bonding through increased surface reactivity. In addition, PVA facilitates stress transfer by forming a bridging network within the matrix. Nevertheless, excessive nanofiber content leads to agglomeration-induced porosity and weak interfacial zones, resulting in reduced compressive strength.
- Considering both mechanical performance and high-temperature resistance, a nanofiber content of 0.5–1 wt% provides the most balanced performance. However, this optimum range reflects a trade-off between enhanced ductility and reduced matrix densification, which limits the compressive load-bearing capacity of the composites.
- A limitation of this study is the absence of comparative control systems containing unmodified hemp fibers or BN-only reinforcement, which restricts the ability to isolate the individual contribution of BN modification. In addition, the long-term durability behavior of BN-modified hemp nanofibers under alkaline geopolymer environments was not investigated. Future studies should therefore focus on durability-related performance, including alkali resistance and long-term mechanical property retention, together with comparative benchmark systems for more rigorous mechanistic evaluation.

Recommendations: Further studies should focus on optimizing the dispersion of higher nanofiber ratios (>2%) to prevent agglomeration, investigating long-term durability under real fire conditions, and evaluating the economic feasibility of BN-modified nanofiber production for large-scale applications.

## Figures and Tables

**Table 1:** Materials used and their properties.

Material	Type/Property	Type/Property
Binder	Blast Furnace Slag (BFS)	Waste from Ereğli Iron and Steel Factory
Alkaline Activators	Sodium Hydroxide (NaOH)	Solid form, 98% purity
	Sodium Silicate (Na_2_SiO_3_)	26.48% SiO_2_, 8.28% Na_2_O, 65.24% H_2_O
Fine Aggregate	CEN Standard Sand	Quartz sand conforming to RILEM-Cembureau standard
Reinforcement Materials	Industrial Hemp Fibers	*Cannabis sativa*, 0.5–3.0 mm size, 1.48–1.50 g/cm^3^ density
	Boron Nitride (BN) Powder	99% purity, 50 nm size, Nanotech
	Polyvinyl Alcohol (PVA)	Mw~140.000, Merck
Chemical Modifiers	3-aminopropiltrietoksisilan (APTES)	Merck
	Ethanol	Distilled water
	2,2,6,6-Tetramethylpiperidine 1-oxyl, 2,2,6,6-Tetramethyl-1-piperidinyloxy (TEMPO)	98% purity, Merck
	Sodium Bromide (NaBr)	Analytical grade, Merck
	Sodium Hypochlorite (NaClO)	Commercial, local market

**Table 2:** Geopolymer mortar mixture proportions.

Sample Code	BFS (%)	NaOH Molarity	Curing Temperature (°C)	Curing Time (h)	Nanofiber Ratio (%)	Test Temperature (°C)
PVA-mBN/Hemp-G0	100	12 M	80, 110	24	0	300, 600, 900, 1200
PVA-mBN/Hemp-G-0.5	100	12 M	80, 110	24	0.5	300, 600, 900, 1200
PVA-mBN/Hemp-G-1	100	12 M	80, 110	24	1	300, 600, 900, 1200
PVA-mBN/Hemp-G-2	100	12 M	80, 110	24	2	300, 600, 900, 1200
PVA-mBN/Hemp-G-4	100	12 M	80, 110	24	4	300, 600, 900, 1200

**Table 3:** Ultrasonic pulse velocity (UPV) and compressive strength values of geopolymer composites containing different ratios of PVA-mBN/Hemp nanofiber reinforcement.

Sample Code	UPV—40 mm (km/s)	UPV—160 mm (km/s)	Compressive Strength (MPa)
PVA-mBN/Hemp G0	2.39 ± 0.04	1.99 ± 0.03	40.22 ± 1.12
PVA-mBN/Hemp G0.5	2.15 ± 0.03	1.87 ± 0.04	32.67 ± 1.08
PVA-mBN/Hemp G1	2.08 ± 0.05	1.44 ± 0.06	30.33 ± 1.25
PVA-mBN/Hemp G2	1.93 ± 0.04	1.02 ± 0.08	29.00 ± 1.30
PVA-mBN/Hemp G4	1.53 ± 0.06	0.43 ± 0.05	8.60 ± 0.15

## Data Availability

The data that support the findings of this study are available from the corresponding author upon reasonable request.
